# Social networks and type 2 diabetes: a narrative review

**DOI:** 10.1007/s00125-021-05496-2

**Published:** 2021-06-29

**Authors:** Miranda T. Schram, Willem J. J. Assendelft, Theo G. van Tilburg, Nicole H. T. M. Dukers-Muijrers

**Affiliations:** 1 Department of Internal Medicine, Heart and Vascular Center, Maastricht University Medical Center+, School for Cardiovascular Diseases (CARIM), Maastricht University, Maastricht, the Netherlands; 2 Department of Primary and Community Care, Radboud University Medical Center, Nijmegen, the Netherlands; 3 Department of Sociology, Vrije Universiteit Amsterdam, Amsterdam, the Netherlands; 4 Department of Sexual Health, Infectious Diseases and Environmental Health, Public Health Service South Limburg, Heerlen, the Netherlands; 5 Department of Health Promotion, Care and Public Health Research Institute (CAPHRI), Maastricht University, Maastricht, the Netherlands

**Keywords:** Diabetes complications, Diabetes management, Living alone, Prevention, Review, Social networks, Social support, Type 2 diabetes

## Abstract

It has been known for decades that social networks are causally related to disease and mortality risk. However, this field of research and its potential for implementation into diabetes care is still in its infancy. In this narrative review, we aim to address the state-of-the-art of social network research in type 2 diabetes prevention and care. Despite the diverse nature and heterogeneity of social network assessments, we can draw valuable lessons from the available studies. First, the structural network variable ‘living alone’ and the functional network variable ‘lack of social support’ have been associated with increased type 2 diabetes risk. The latter association may be modified by lifestyle risk factors, such as obesity, low level of physical activity and unhealthy diet. Second, smaller network size and less social support is associated with increased risk of diabetes complications, particularly chronic kidney disease and CHD. Third, current evidence shows a beneficial impact of social support on diabetes self-management. In addition, social support interventions were found to have a small, favourable effect on HbA_1c_ values in the short-term. However, harmonisation and more detailed assessment of social network measurements are needed to utilise social network characteristics for more effective prevention and disease management in type 2 diabetes.

**Graphical abstract:**

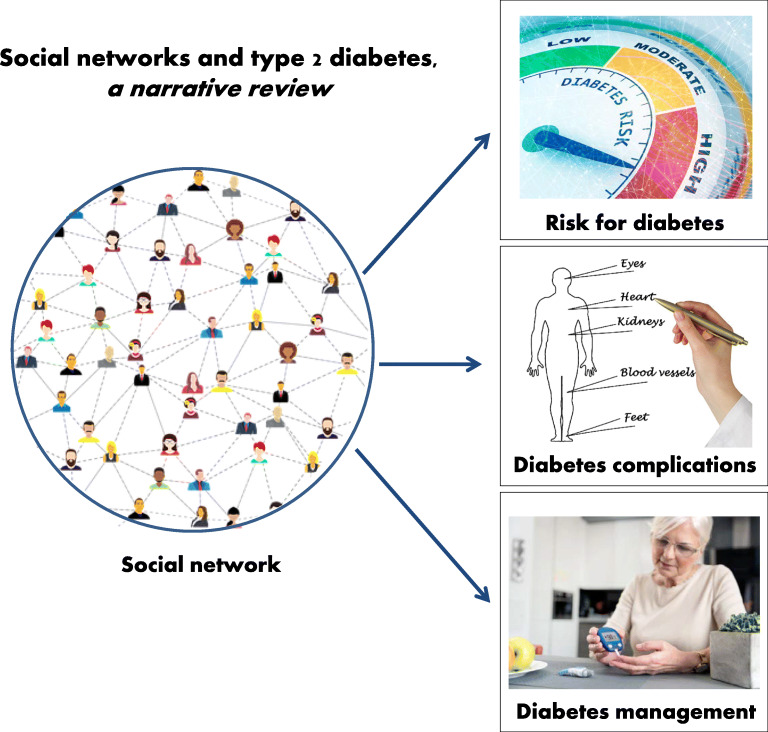

**Supplementary Information:**

The online version contains a slideset of the figures for download available at 10.1007/s00125-021-05496-2.

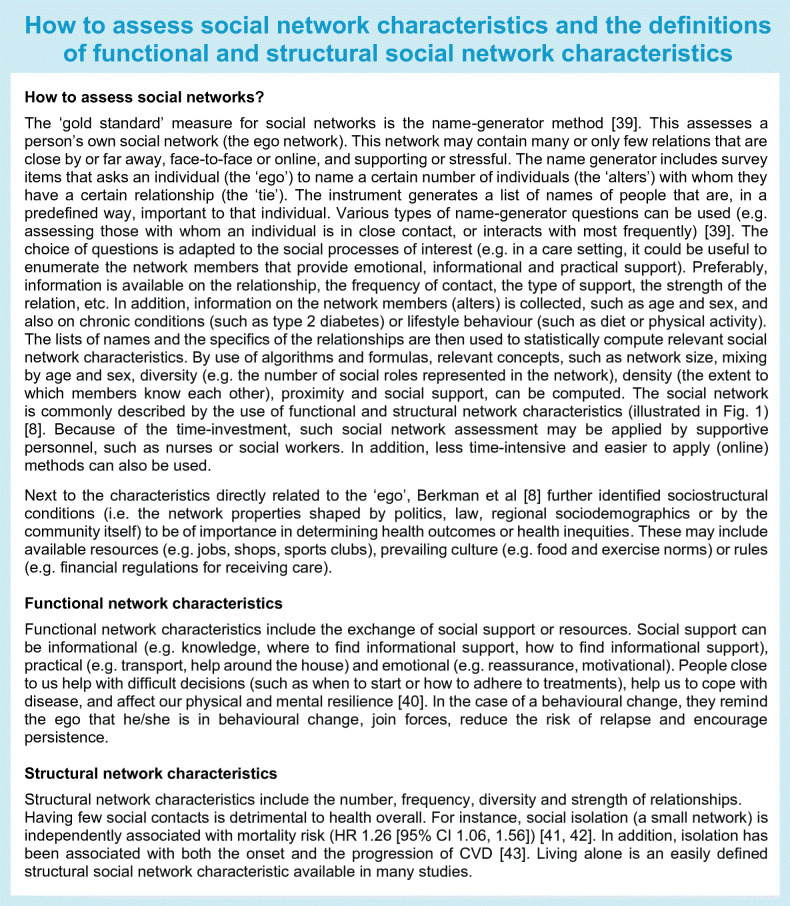



## Introduction

The rising prevalence of type 2 diabetes globally urges the diabetes community to find new solutions for the prevention of type 2 diabetes and its severe complications. However, employing effective and sustainable interventions for type 2 diabetes risk factors, such as obesity and lack of physical activity, appears to be challenging. In general, lifestyle changes are adopted by individuals so long as there is intensive coaching and supervision by health professionals but fade away when this is stopped. Engaging non-professional peers within an individual’s social network may offer an additional opportunity to bring about and maintain behavioural change. Prevention strategies that utilise social integration and social support may prove promising in type 2 diabetes prevention and care [1, 2].

The recent Coronavirus disease-2019 (COVID-19) pandemic has added another dimension to this concept; measures for social distancing were introduced in societies, including keeping physical distance from others and avoiding gathering in large groups. The economic and social drawbacks of social distancing that individuals and society are struggling with include reduced productivity, loneliness and the loss of practical assistance, as well as lack of informational, financial and emotional support.

Given the above, we need to better understand the importance of social contacts for health in general and for type 2 diabetes and diabetes care in particular [3]. In this narrative review we aim to address the state-of-the-art of social network research in type 2 diabetes prevention and care. We will explicitly address objectively measured social relationships, but exclude loneliness, which is addressed elsewhere [4, 5] and refers to a subjective sense of lack of number and quality of social relationships [6].

First, we will address the aetiology that may link social networks to type 2 diabetes. Second, we will summarise the literature on the association between social network characteristics and incidence of type 2 diabetes. Third, evidence on the impact of the social network on the course of diabetes will be conferred. Fourth, the association of the social network with disease management will be discussed.

## The aetiology that may link social networks to diabetes

**Fig. 1:**
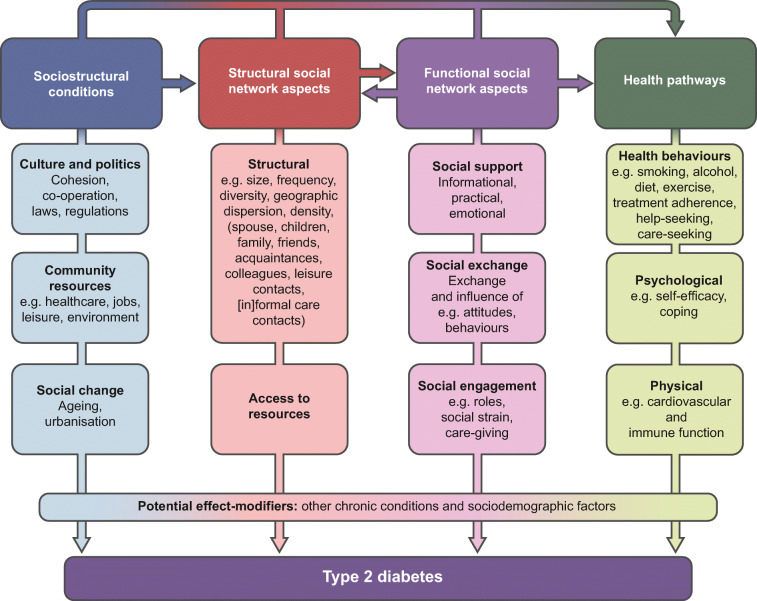
Theoretical model of social networks and type 2 diabetes. A discrimination can be made between functional and structural characteristics of the social network. Functional characteristics involve a qualitative scoring of the individual’s social relationships (also referred to as social support). This includes the individual’s own perception, degree of satisfaction and realisation of the support from others. Structural characteristics involve a quantitative scoring of the availability and number of people around the individual. Figure based on information from Berkman et al [8]. This figure is available as part of a downloadable slideset

Social networks can be defined as the web of social relationships that surround an individual, connecting that individual with family, friends, colleagues, neighbours and potentially also health professionals. Social networks are an essential aspect of life, serving important social, psychological and behavioural functions. Information on how to assess social network characteristics and the definitions of functional and structural social network characteristics can be found in the Text box/Fig. 1. Research into the role of social networks in type 2 diabetes started in the early 2000s. Two main hypotheses on its aetiology have been proposed: the stress-buffering or stress-exacerbating hypothesis; and the social contagion hypothesis or behavioural hypothesis [7, 8]. Regarding the first hypothesis, stress in itself is able to affect physiological processes. Stress may for instance result in weight gain or dysfunction of the hypothalamic–pituitary–adrenal axis, which in turn can lead to type 2 diabetes. In particular, the crosstalk between autonomic, endocrine and inflammatory pathways may prove important. However, the lack of experimental data to support these theories should be noted. In turn, social support may alleviate stress and thus buffer the adverse physiological changes, thereby preventing or delaying the onset of type 2 diabetes. Conversely, negative aspects of relationships (i.e. so-called social strain) may cause stress and thus adversely affect health outcomes. Second, the social contagion hypothesis refers to the tendency of individuals to adopt behaviours from others in their social network. For instance, healthy or unhealthy lifestyle behaviour may be promoted via key people within a social network. This hypothesis is exemplified by the ground-breaking work of Christakis and Fowler, using data from the Framingham Heart Study [9]. Their study showed that obesity spreads through social networks and confirmed that connected people may share lifestyle factors (e.g. physical activity, diet) or may experience simultaneous events that cause them to gain or lose weight simultaneously. Importantly, the authors’ observations suggested that the social network also had an effect on its own, revealing a process involving person-to-person spread of obesity through social relations.

## Social networks in type 2 diabetes

**Fig. 2:**
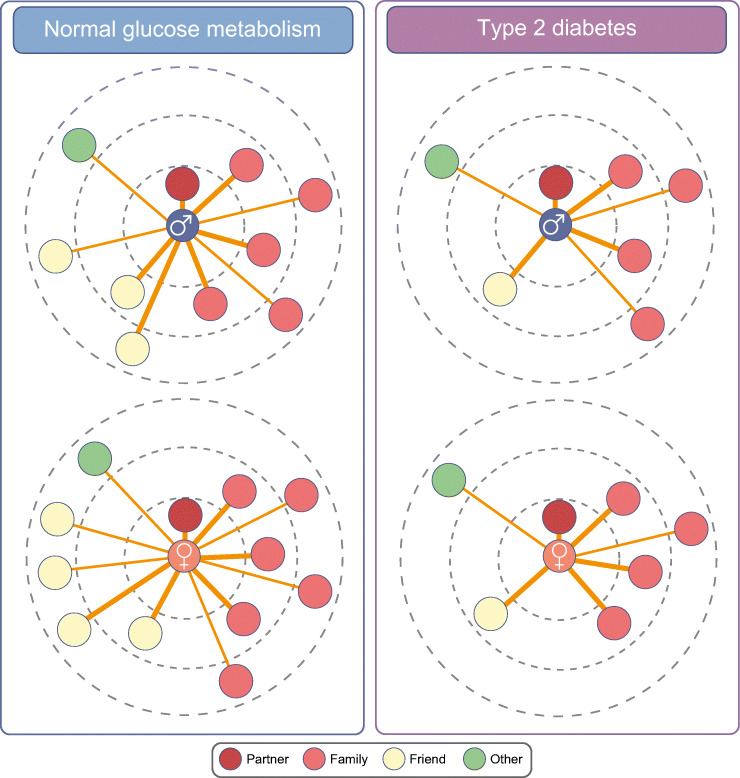
Structural social network characteristics in diabetes. The composition of structural network characteristics in men and women with and without type 2 diabetes, according to real-life data from the population-based Maastricht Study is shown. Blue (men) and pink (women) circles represent the ego, dark-red circles represent the partner, light-red circles represent family members, yellow circles represent friends and the green circles represent another type of contact. The dashed circles represent geographical living distance: inner circle, household; middle circle, walking distance; and outer circle, more than walking distance. The lines between the ego and network member represent the frequency of contact: bold ‘ties’ represent daily/weekly contact, whilst non-bold ‘ties’ represent monthly or less contact. Figure adapted from Brinkhues et al [11] under the terms of the Creative Commons Attribution 4.0 International License (http://creativecommons.org/licenses/by/4.0/), which permits unrestricted use, distribution, and reproduction in any medium. This figure is available as part of a downloadable slideset

Clear differences in specific aspects of the social network have been demonstrated when comparing men and women. For instance, men are known to have smaller social networks than women [10]. This also applies to men and women with type 2 diabetes (seven vs eight network members) [11]. In addition, women receive more emotional support via their social network than men, whereas men are thought to receive major support from their partner [10]. Figure 2 depicts the composition of structural network characteristics in men and women with and without type 2 diabetes, according to data from the population-based Maastricht Study [11]. The number of circles shown in Fig. 2 represents the mean network size for men and women with normal glucose metabolism and with type 2 diabetes. Please note the smaller network size for men vs women with normal glucose metabolism and for individuals with type 2 diabetes compared with those with normal glucose metabolism.

## Social networks and development of type 2 diabetes

Here, we summarise evidence of social networks as risk factor for type 2 diabetes. We focus on longitudinal data to address the temporality of these associations, and in particular on the social network constructs ‘living alone’ and ‘social support’*.* These two constructs have a relatively uniform definition and operationalisation across the various empirical studies and therefore represent reproducible social network measures, while other measures used were too diverse in methods or nature to compile meaningfully. Although the evidence is relatively scarce, we found several well-designed large-scale studies on this topic (see Table 1).

**Table 1:** Overview of literature on the association between social network characteristics and type 2 diabetes risk

Study	Study name and population	Follow-up duration (years)	*N*	(Incident) T2D cases (*n*)	Mean age or age range (years)	Measures of social network	Measurement of diabetes	Main results	Study limitations
Cross-sectional study design									
Brinkhues (2017) [11]	The Maastricht Study. Population-based, oversampled diabetes.	NA	2861	808	40–75	Various structural and functional network characteristics	OGTT	Small network size associated with newly and previously diagnosed diabetes. In women, larger distance, more household members and fewer friends associated with newly diagnosed and known diabetes. Lack of social participation associated with diabetes in men and women. Living alone associated with diabetes in men only. Less support in men and women with new and previous diabetes vs those with NGT.	Relatively healthy population
Jones (2016) [35]	NHATS. Medicare representative survey, USA.	NA	6942	Unknown	65–94	Number of important people (0–5)	Registry	Diabetes associated with smaller network.	Study focused on multimorbidity; not diabetes specific
Hempler (2013) [36]	Part of Danish health and morbidity survey. Diabetes clinic vs web panel, diabetes vs general population.	NA	Diabetes clinic: 1084; web panel: 1491; general population: 15,165	2575	Diabetes clinic: 64; web panel: 60; general population: 50	Social structure (household, contact family/friends) and function (count on others help)	Clinic or self-report	Living alone (diabetes risk: OR 1.75–1.64), meeting family less than once per month (diabetes risk: OR 1.78–2.35).	Limited assessment of social network
Longitudinal study design									
Hendryx (2020) [18]	Women’s Health Initiative. Postmenopausal women from 40 US clinical centres.	14	139,924	19,240	50–79	Social support (9 item scale), social network (range: 0–5), social strain (4 item scale)	Self-reported new diagnosis of T2D treated with insulin or oral drugs	Social support is protective for incident diabetes. BMI, PA and depression mediate association between support and incident diabetes.	Limited assessment of social network
Lukaschek (2017) [12]	MONICA/KORA. General population.	14	6839	551	25–74	SNS (one item), living alone, SNI (dichotomous)	Self-reported or validated by physicians	Low SNS associated with doubled diabetes risk, partly via social isolation and living alone, but only in men (living alone in men, HR 1.62 [95% CI 1.22, 2.15]).	A construct of social network (SNI) was used; limited assessment of social network
Laursen (2017) [19]	ELSA. General population.	7.7	7662	804	50–91	Social relationships (spouse, children, other immediate family, friends; score 0–4), social support per relationship (score 0–36), social strain (3 item)	Self-reported or screen-detected T2D	Higher support and low strain are associated with lower diabetes risk, dependent on BMI and health behaviour (smoking, alcohol consumption and PA).	Limited assessment of social network
Altevers (2016) [20]	MONICA/KORA. General population.	15.5	8952	904	30–74	SNI (dichotomous)	Self-reported T2D validated with physicians’ records	Poor vs good structural support is associated with increased diabetes risk in men and women, and only in men after adjustment (age, survey, smoking, alcohol, PA, parent with diabetes, BMI, hypertension, dyslipidaemia) (men, HR 1.31 [95% CI 1.11, 1.55]; women HR 1.10 [95% CI 0.88, 1.37).	A construct of social network (SNI) was used; limited assessment of social network
Hilding (2015) [13]	Stockholm diabetes prevention programme. General population.	8–10	4963	143	35–56	AVSI index (6 items; score 0–12), civil status (partner, yes/no), participation in social activity (2 items; dichotomous)	OGTT	High AVSI is associated with low diabetes risk in women, partly dependent on BMI, PA and smoking. In men, high AVSI is associated with high diabetes risk. Social participation decreases diabetes risk in men and women. Having a partner is protective for diabetes in men only: for T2D, OR 0.57 (95% CI 0.33, 0.97) and OR 0.61 (95% CI 0.34, 1.08) in age- and multi-adjusted models, respectively.	A construct of social network (AVSI) was used; Limited assessment of social network
Meisinger (2009) [14]	MONICA/KORA Augsburg Cohort Study. General population.	10.9	8804	673	35–74	Living alone assessed by one question	Questionnaire and clinical validation	Living alone is a risk factor for diabetes in men, not women: HR 1.66 (95% CI 118, 2.34) vs HR 0.86 (95% CI 0.58, 1.26).	Limited assessment of social network (only living alone)
Strodl (2006) [15]	Australian women’s health survey. Women from the general population.	3	10,300	243	70–74	DSSI (11 items; 2 factors: social satisfaction and social interaction), marital status	Participants asked, ‘did a doctor inform you that you have diabetes?’ (no distinction between T1D or T2D)	Moderate social support predicts new diabetes in elderly women in univariate analysis.	A construct of social network (DSSI) was used; Limited assessment of social network
Lidfeldt (2005) [16]	Study name: NA. Women with IGT from population registry.	2.5	461	55	50–64	Living alone, marital status	OGTT to assess T2D	Living alone is a risk factor for T2D (age-adjusted OR 2.47 [95% CI 1.06, 5.71]) but depends on smoking status (fully adjusted OR 2.07 [95% CI 0.62, 6.88]).	Limited assessment of social network (only living alone and marital status)
Kumari (2004) [17]	Whitehall II study. Occupational cohort study, London (UK).	10.5 (4 years follow-up moments^a^)	10,308	361	35–55	Social support (Close Persons Questionnaire), questions derived by Berkman and Syme. The network measures were summarised on a scale measuring the network beyond the household.	Self-reported, physician-diagnosed or OGTT	Effort–reward imbalance is associated with incident diabetes in men.	A construct of social network (Close Persons Questionnaire) was used; Limited assessment of social network
Norberg (2007) [37]	Health survey. Occupational population, Umea (Sweden); age- and sex-matched nested case-referent study	5.4 ± 2.6	584	191	Cohort 1: 40; cohort 2: 50; cohort 3: 60	Social network and emotional support (interview schedule for social interaction), social integration (AVSI, 7 questions), attachment (AVAT, 7 questions)	Medical registry-based diagnosis of T2D	Low AVAT associated with incident T2D in women (weak AVAT: OR 3.0 [95% CI 1.3, 7.0]).	A construct of social network (AVSI, AVAT) was used; Limited assessment of social network
Hill (2014) [38]	Health and retirement study, USA Representative sample of ageing population	4	5681	430	68	Social relationships (spouse, children, other immediate family, friends; score 0–4), social support per relationship (score 0–36), social strain (3 items)	Self-reported	Negative friend support increases the risk for diabetes (OR 1.31 [95% CI 1.07, 1.62]).	Limited assessment of social network

^a^Follow-up data were collected at four different points in time and these data were merged (no continuous follow-up)

AVAT, availability of attachment; AVSI, availability of social integration; DSSI, Duke social support index; IGT, impaired glucose tolerance; NA, not applicable; NGT, normal glucose tolerance; NHATS, National Health and Aging Trends Study; PA, physical activity; SNI, Social Network Index; T1D, type 1 diabetes; T2D, type 2 diabetes

First, living alone has been associated with an increased type 2 diabetes risk among men [12–14] but this may depend on lifestyle factors like smoking and dietary habits in women [15, 16]. Effect estimates range from a 1.39- to 1.66-fold increase in risk of type 2 diabetes for men living alone, which may be similar to the imposed risk by well-known cardiovascular risk factors, like hypertension, dyslipidaemia or obesity [17].

Second, various measures of social support have been prospectively associated with type 2 diabetes risk. An extensive study by Hendryx et al [18] within the Women’s Health Initiative showed that lack of social support increases type 2 diabetes risk. Moreover, women with the highest level of social support had a lower type 2 diabetes risk (HR 0.93) [18]. In line with these results, the English Longitudinal Study of Ageing (ELSA) study [19] showed an increased type 2 diabetes risk among individuals with low levels of social support. Of particular interest, some of the above associations were mediated by lifestyle factors, such as smoking, physical activity, BMI and depression. Furthermore, the MONICA/KORA study [20] showed similar results: low social support was associated with an increased type 2 diabetes risk in men but not in women.

In summary, results clearly show that living alone has been associated with an increased type 2 diabetes risk, particularly in men. Any such association in women may be mediated by lifestyle risk factors. Furthermore, lack of social support also increases type 2 diabetes risk, with this association being dependent on lifestyle risk factors. Although the constructs ‘living alone’ and ‘social support’ do not reflect the complexity of social networks and only partially provide targets for intervention, they do illustrate the importance of social networks for type 2 diabetes risk. However, the lack of experimental studies and the possibility of reverse causation should be taken into account when interpreting these results.

## Social networks and diabetes complications

Next to diabetes itself, social networks may be associated with type 2 diabetes complications. An overview of the available literature on this topic is presented in Table 2. Cross-sectional name-generator data from The Maastricht Study have shown that type 2 diabetes patients with smaller social networks have a higher prevalence of self-reported macrovascular complications such as myocardial infarction and stroke [21]. Also, a lack of network diversity (assessed as a high percentage of family members and a low percentage of friends within the social network) was associated with macrovascular complications, while no association was found with emotional, informational or practical support. In addition, smaller social networks and less informational support was related to microvascular complications in women (such as albuminuria, retinopathy and peripheral neuropathy) but not in men. The observed sex difference may be due to different coping strategies between men and women. Furthermore, a Japanese study observed that higher levels of social support and a highly connected social network was associated with a lower prevalence of diabetic nephropathy [22].

**Table 2:** Overview of literature on the association between social network characteristics and type 2 diabetes complications

Study	Study name and population	Follow-up duration (years)	*N*	Incident cases (*n*)	Mean age or age range (years)	Measurement of social network	Diabetes complication	Measurement of diabetes	Main results
Cross-sectional study design									
Brinkhues (2018) [21]	The Maastricht Study. T2D from general population.	NA	797	NA	63	Name generator	Macro- and microvascular complications	OGTT and/or medication	Small network, high % family members and low % friends is associated with CVD (ORs 1.00–1.22). In women, small network and less informational support is associated with microvascular complications (ORs 1.02–1.71).
Ninomiya (2018) [22]	Study name: NA. T2D from general population, Osaka (Japan).	NA	123 with diabetic nephropathy, 220 without	NA	65	Social network and social support (SNI and ESSI, dichotomised)	eGFR and spot urine to assess micro/macroalbuminuria and albumin/creatine ratio. Retinopathy according to ophthalmologist. Diabetic neuropathy (symptoms, absence of tendon reflex, reduced vibration)	According to Japanese Diabetes Society, age 30–80 years	High connection of social network (OR per SD, 0.35–0.87) and more social support (OR per SD 0.38–0.96) were associated with a reduced risk of the presence of diabetic nephropathy.
Longitudinal study design									
Miao Jonasson (2020) [24]	Women’s Health Initiative. Postmenopausal women.	12.79	5262	672	64	Social support (9 questions on how often participants could access each type of support), network size (size index 0–5 based on married/club/religious ties, number of relatives)	CHD (myocardial infarction or CHD death)	Self-reported	Married/intimate relationship decreased CHD risk (HR 069–0.97). Q3 of social support (high social support) associated with lower CHD risk. Indication that health behaviours (e.g. PA and healthy diet) may be mediators of associations between social network size and risk of CHD.
Dunkler (2015) [23]	ONTARGET. Clinical trial of T2D with normo- or microalbuminuria.	5.5	6972	2182	55+	SNS: participant’s physical social network, defined as the number of social interactions and personal relationships, was assessed at baseline by 4 questions quantifying the number of people one regularly interacts with. To quantify the size of a participant’s social network, a summary score of the 4 questions was derived	CKD	Not specified	Risk of CKD was 11% lower in T3 of SNS compared with the T1 (OR [95% CI] for CKD, T3 vs T1: 0.86 [0.81, 0.97]).

CKD, chronic kidney disease; ESSI, Enhancing Recovery in Coronary Heart Disease; NA, not applicable; PA, physical activity; Q3, quartile 3; SNI, social network index; T1, tertile 1; T2D, type 2 diabetes; T3, tertile 3

Longitudinal evidence on the association between social network and diabetes complications comes from two large-scale studies: an observational study from the Ongoing Telmisartan Alone and in Combination with Ramipril Global Endpoint Trial (ONTARGET) [23]; and the Women’s Health Initiative [24]. Both used a relatively generic measure of social network characteristics. ONTARGET investigated the association of the social network score (SNS) with chronic kidney disease (CKD) in high-risk individuals with type 2 diabetes [23]. An 11% reduction in CKD risk was observed when comparing the third with the first tertile of the SNS over 5.5 years of follow-up. This association was independent of lifestyle and cardiovascular risk factors. The authors suggested that a tight social network helps in the (self-)management of diabetes and therefore prevents complications. The Women’s Health Initiative focused on the association of social support and smaller network size with incident CHD in postmenopausal women with type 2 diabetes [24]. Being married or in an intimate relationship appeared to protect against the development of CHD (HR 0.82). In addition, being in the third quartile of social network size was associated with a lower CHD risk, as compared with being in the first quartile. Furthermore, this study indicated that health behaviours, such as physical activity and healthy diet, might be mediators of the associations between social network size and risk of CHD.

In summary, there is cross-sectional and longitudinal evidence that structural (e.g. smaller network size) and functional (e.g. less support) social network measures are associated with an increased risk of type 2 diabetes complications. The potential mediating role of lifestyle factors was inconsistent across studies. The study findings outlined above demonstrate the paucity of high-quality studies on this topic but also reveal the potential of detailed social network assessment in the identification of targets for the prevention of type 2 diabetes complications. There is also a lack of experimental data by which to assess true causality and the possibility of reverse causation (e.g. reduced functionality due to diabetes complications could also reduce social network size).

## Social networks and diabetes management

Social networks may also impact treatment of individuals with type 2 diabetes. Nowadays, self-management is a cornerstone of diabetes care. Moreover, there is increasing recognition that self-management can be seen as a social process that involves social networks and personal communities and requires the mobilisation of social resources [25]. Increasing evidence underlines the supportive role of healthcare professionals in diabetes management and education. Also, the role of peer support (e.g. via patient participation groups) has also gained attention. Nevertheless, the role of informal, interpersonal relationships in diabetes care, and the support that is provided by an individual’s personal network, has been understudied. In 2005, van Dam et al [26] systematically reviewed six RCTs that applied a variety of social support interventions, but concluded that no meaningful comparison or assessment of potential mechanisms was possible due to the diverse nature of the studies. A systematic review of observational studies experienced the same difficulties [27]. Despite these limitations, the authors concluded that there was some evidence for a beneficial association between social support and glycaemic control. Their practical recommendation was to explore the presence of informational support within routine diabetes care.

The lack of structured and standardised social network measures in literature led to a qualitative meta-synthesis being undertaken [25]. This analysis identified three key social network mechanisms in diabetes management: (1) sharing knowledge and experiences in a personal community; (2) accessing and mediation of resources; and (3) awareness of and ability to deal with network relationships (which is required for self-management support). These key mechanisms may provide the essential background for applying theory-based interventions in diabetes care and were used to guide later studies. For instance, one such study, by Koetsenruijter et al [28], evaluated the role of social support and self-management capabilities in type 2 diabetes using state-of-the-art measures of social support. In this study, which was embedded within the EU-WISE project, higher educational level and income were negatively related to self-management capabilities but larger informational and emotional support networks showed a positive association with self-management capabilities. In the same study population, social support from individuals and community organisations was associated with better health status and health-related behaviours, especially in low-income populations [29]. Finally, a Korean study in women with type 2 diabetes found that social support positively affected self-efficacy of diabetes management, including diet, frequency of exercise and symptom management [30].

In summary, we conclude that the limited evidence available illustrates that social support may promote self-management of type 2 diabetes. Many lacunes remain to provide clear advice on social network interventions in type 2 diabetes management.

## Potential for social networks in the prevention of type 2 diabetes

As described by Christakis and Fowler [9], risk factors for the development of type 2 diabetes, such as obesity, may spread via the social network. Their study also showed that specific ties, such as mutual friendships, same-sex friends or spouses, are important with regard to the spread of obesity. Social closeness appeared to be more important than geographical distance. Further analyses on the Framingham Heart Study also showed that obesity and type 2 diabetes within a person’s social network were associated with an increased risk of developing type 2 diabetes, and that these associations were mediated by shared health behaviours, such as diet and physical activity [31]. Additional support for these observations comes from Bot et al [32], who reported that adults with larger and denser social networks have healthier lifestyles, including higher levels of vegetable consumption and physical activity and lower levels of sedentary behaviour.

A recent meta-analysis [33] summarised RCTs that assessed the effectiveness of social network interventions on HbA_1c_, quality of life and social support. Despite the highly heterogeneous, insufficiently detailed interventions and study designs, a small favourable effect of social support was found for HbA_1c_ at 3 months (a mean 0.25 percentage points [1.55 mmol/mol] decrease). The authors conclude that there is a need for clear theories and hypotheses that will enable firmer assessment of the potential of social support interventions in diabetes care.

## Conclusions

In summary, previous research opens new doors for using social network interventions in type 2 diabetes prevention, prevention of type 2 diabetes complications and diabetes self-management. Social network ties are important in determining one’s perception, behaviour and norms regarding health behaviour. However, intervention research using the social network in the implementation of lifestyle interventions or disease management is only just starting to develop. Studies that specifically assess the additional value of involving a person’s social network in interventions are urgently needed. A recent critical synthesis on the integration of social network properties in obesity prevention may provide some first clues for designing future interventions [34].

Research into the impact of social networks on health is a relatively new field of research. This is demonstrated by the large variety of social network concepts and measurements that have been used in previous studies, which hampers the ability to draw firm conclusions and lessons for clinical practice. There is a clear need for harmonisation of social network measurements and the collection of detailed high-quality data that enable replication of findings. Obviously, we lack specific knowledge about which network aspects play a role, alone and in combination, in type 2 diabetes onset, type 2 diabetes management and care, and in which contexts they apply. More explicit knowledge is needed to enable development of specific interventions.

However, despite these methodological difficulties, there are lessons learned. First, there is convincing longitudinal evidence that living alone, particularly in men, and lack of social support for both men and women are associated with an increased type 2 diabetes risk. In scientific studies these social characteristics clearly precede the development of type 2 diabetes and may thus serve as an indicator for increased type 2 diabetes risk. Second, lack of structural or functional social support is associated with an increased risk of severe type 2 diabetes complications, such as CKD and CHD. Third, although the evidence base may be less extensive, social support may help to implement or improve diabetes self-management. Finally, these associations may, in part, be mediated by lifestyle risk behaviours, such as obesity, lack of physical activity and an unhealthy diet.

## Supplementary Information

ESM (PPTX 317 kb)

## Abbreviations

CKD: Chronic kidney disease
ONTARGET: Ongoing Telmisartan Alone and in Combination with Ramipril Global Endpoint
SNS: Social network score

## References

[1] Valente TW (2010). Social networks and health.

[2] Haenay CA (2008). Health behaviour and health education. Theory, research and practice.

[3] Brooks SK, Webster RK, Smith LE (2020). The psychological impact of quarantine and how to reduce it: rapid review of the evidence. Lancet.

[4] Christiansen J, Larsen FB, Lasgaard M (2016). Do stress, health behavior, and sleep mediate the association between loneliness and adverse health conditions among older people?. Soc Sci Med.

[5] Christiansen J, Lund R, Qualter P, Andersen CM, Pedersen SS, Lasgaard M (2021). Loneliness, social isolation, and chronic disease outcomes. Ann Behav Med.

[6] Gierveld JDJ, Tilburg TGV, Dykstra PA, Vangelisti AL, Perlman D (2018). New ways of theorizing and conducting research in the field of loneliness and social isolation. The Cambridge handbook of personal relationships.

[7] Cohen S (2004). Social relationships and health. Am Psychol.

[8] Berkman LF, Kawachi I, Glymour MM (2014). Social epidemiology.

[9] Christakis NA, Fowler JH (2007). The spread of obesity in a large social network over 32 years. N Engl J Med.

[10] Fuhrer R, Stansfeld SA (2002). How gender affects patterns of social relations and their impact on health: a comparison of one or multiple sources of support from “close persons”. Soc Sci Med.

[11] Brinkhues S, Dukers-Muijrers N, Hoebe C (2017). Socially isolated individuals are more prone to have newly diagnosed and prevalent type 2 diabetes mellitus - the Maastricht study. BMC Public Health.

[12] Lukaschek K, Baumert J, Kruse J, Meisinger C, Ladwig KH (2017). Sex differences in the association of social network satisfaction and the risk for type 2 diabetes. BMC Public Health.

[13] Hilding A, Shen C, Ostenson CG (2015). Social network and development of prediabetes and type 2 diabetes in middle-aged Swedish women and men. Diabetes Res Clin Pract.

[14] Meisinger C, Kandler U, Ladwig KH (2009). Living alone is associated with an increased risk of type 2 diabetes mellitus in men but not women from the general population: the MONICA/KORA Augsburg Cohort Study. Psychosom Med.

[15] Strodl E, Kenardy J (2006). Psychosocial and non-psychosocial risk factors for the new diagnosis of diabetes in elderly women. Diabetes Res Clin Pract.

[16] Lidfeldt J, Nerbrand C, Samsioe G, Agardh CD (2005). Women living alone have an increased risk to develop diabetes, which is explained mainly by lifestyle factors. Diabetes Care.

[17] Kumari M, Head J, Marmot M (2004). Prospective study of social and other risk factors for incidence of type 2 diabetes in the Whitehall II study. Arch Intern Med.

[18] Hendryx M, Nicholson W, Manson JE (2020). Social relationships and risk of type 2 diabetes among postmenopausal women. J Gerontol B Psychol Sci Soc Sci.

[19] Laursen KR, Hulman A, Witte DR, Terkildsen Maindal H (2017). Social relations, depressive symptoms, and incident type 2 diabetes mellitus: the English longitudinal study of ageing. Diabetes Res Clin Pract.

[20] Altevers J, Lukaschek K, Baumert J (2016). Poor structural social support is associated with an increased risk of type 2 diabetes mellitus: findings from the MONICA/KORA Augsburg cohort study. Diabet Med.

[21] Brinkhues S, Dukers-Muijrers N, Hoebe C (2018). Social network characteristics are associated with type 2 diabetes complications: the Maastricht study. Diabetes Care.

[22] Ninomiya H, Katakami N, Matsuoka TA (2018). Association between poor psychosocial conditions and diabetic nephropathy in Japanese type 2 diabetes patients: a cross-sectional study. J Diabetes Investig.

[23] Dunkler D, Kohl M, Heinze G (2015). Modifiable lifestyle and social factors affect chronic kidney disease in high-risk individuals with type 2 diabetes mellitus. Kidney Int.

[24] Miao Jonasson J, Hendryx M, Shadyab AH (2020). Social support, social network size, social strain, stressful life events, and coronary heart disease in women with type 2 diabetes: a cohort study based on the Women’s Health Initiative. Diabetes Care.

[25] Vassilev I, Rogers A, Kennedy A, Koetsenruijter J (2014). The influence of social networks on self-management support: a metasynthesis. BMC Public Health.

[26] van Dam HA, van der Horst FG, Knoops L, Ryckman RM, Crebolder HF, van den Borne BH (2005). Social support in diabetes: a systematic review of controlled intervention studies. Patient Educ Couns.

[27] Stopford R, Winkley K, Ismail K (2013). Social support and glycemic control in type 2 diabetes: a systematic review of observational studies. Patient Educ Couns.

[28] Koetsenruijter J, van Eikelenboom N, van Lieshout J (2016). Social support and self-management capabilities in diabetes patients: an international observational study. Patient Educ Couns.

[29] Koetsenruijter J, van Lieshout J, Lionis C (2015). Social support and health in diabetes patients: an observational study in six European countries in an era of austerity. PLoS One.

[30] Park H, Kim MT (2012). Impact of social role strain, depression, social support and age on diabetes self-efficacy in Korean women with type 2 diabetes. J Cardiovasc Nurs.

[31] Raghavan S, Pachucki MC, Chang Y (2016). Incident type 2 diabetes risk is influenced by obesity and diabetes in social contacts: a social network analysis. J Gen Intern Med.

[32] Bot SD, Mackenbach JD, Nijpels G, Lakerveld J (2016). Association between social network characteristics and lifestyle Behaviours in adults at risk of diabetes and cardiovascular disease. PLoS One.

[33] Spencer-Bonilla G, Ponce OJ, Rodriguez-Gutierrez R (2017). A systematic review and meta-analysis of trials of social network interventions in type 2 diabetes. BMJ Open.

[34] Serrano Fuentes N, Rogers A, Portillo MC (2019). Social network influences and the adoption of obesity-related behaviours in adults: a critical interpretative synthesis review. BMC Public Health.

[35] Jones SM, Amtmann D, Gell NM (2016). A psychometric examination of multimorbidity and mental health in older adults. Aging Ment Health.

[36] Hempler NF, Ekholm O, Willaing I (2013). Differences in social relations between persons with type 2 diabetes and the general population. Scand J Public Health.

[37] Norberg M, Stenlund H, Lindahl B, Andersson C, Eriksson JW, Weinehall L (2007). Work stress and low emotional support is associated with increased risk of future type 2 diabetes in women. Diabetes Res Clin Pract.

[38] Hill PL, Weston SJ, Jackson JJ (2014). Connecting social environment variables to the onset of major specific health outcomes. Psychol Health.

[39] McCallister L, Fischer CS (1978). A procedure for surveying personal networks. Sociol Methods Res.

[40] Ozbay F, Johnson DC, Dimoulas E, Morgan CA, Charney D, Southwick S (2007). Social support and resilience to stress: from neurobiology to clinical practice. Psychiatry (Edgmont).

[41] Elovainio M, Hakulinen C, Pulkki-Raback L (2017). Contribution of risk factors to excess mortality in isolated and lonely individuals: an analysis of data from the UK Biobank cohort study. Lancet Public Health.

[42] Holt-Lunstad J, Smith TB, Baker M, Harris T, Stephenson D (2015). Loneliness and social isolation as risk factors for mortality: a meta-analytic review. Perspect Psychol Sci.

[43] Valtorta NK, Kanaan M, Gilbody S, Ronzi S, Hanratty B (2016). Loneliness and social isolation as risk factors for coronary heart disease and stroke: systematic review and meta-analysis of longitudinal observational studies. Heart.

