# Supplementary material for: Social networks and type 2 diabetes: a narrative review
Source: Diabetologia. 2021 Jun 29;64(9):1905–16. doi: 10.1007/s00125-021-05496-2 (PMC8241411; doi:10.1007/s00125-021-05496-2)
Supplement: Supplementary file 1 — (PPTX 317 kb) [file 125_2021_5496_MOESM1_ESM.pptx]

## Slide 1
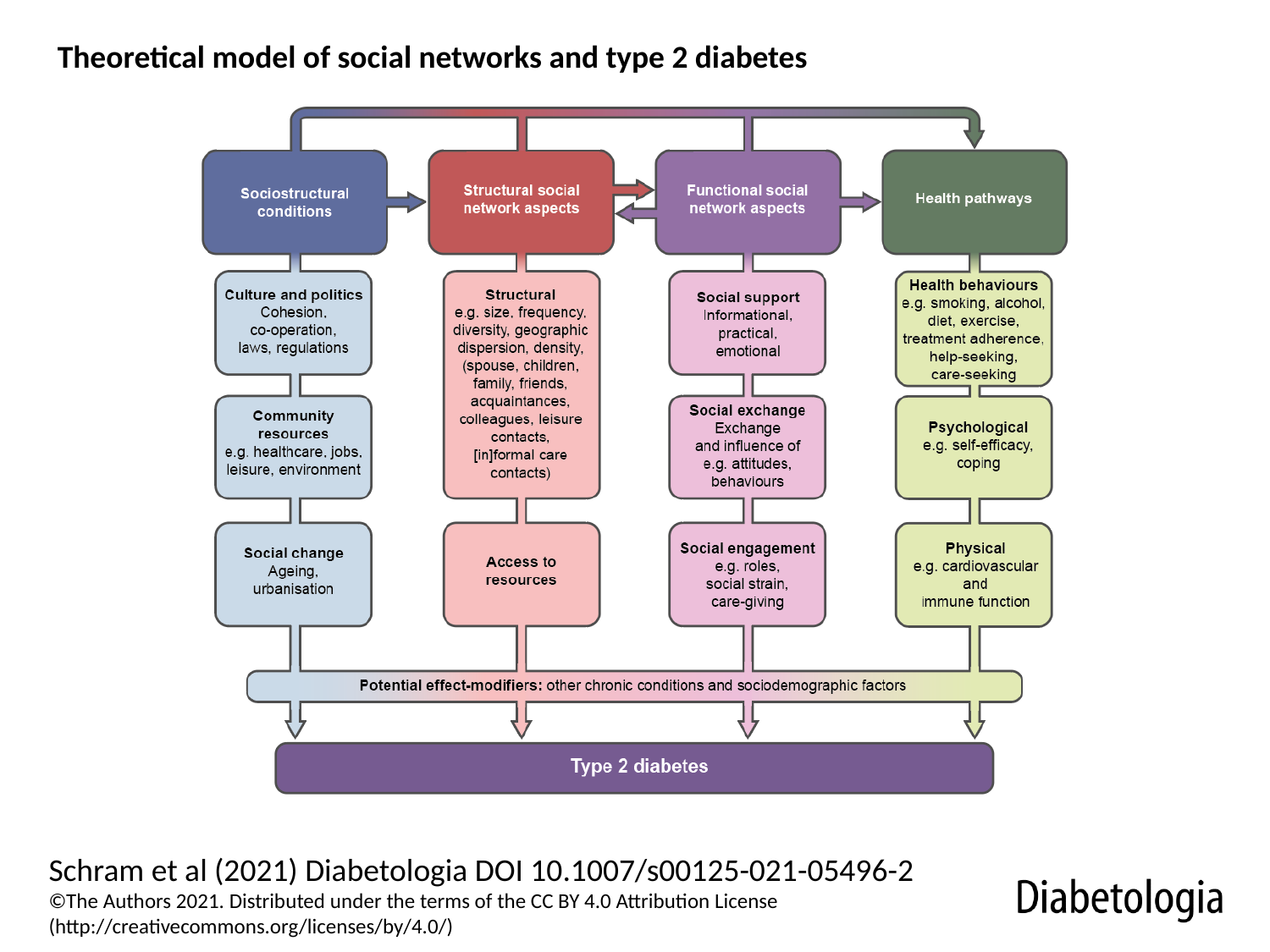

Theoretical model of social networks and type 2 diabetes
Schram et al (2021) Diabetologia DOI 10.1007/s00125-021-05496-2
©The Authors 2021. Distributed under the terms of the CC BY 4.0 Attribution License (http://creativecommons.org/licenses/by/4.0/)

## Slide 2
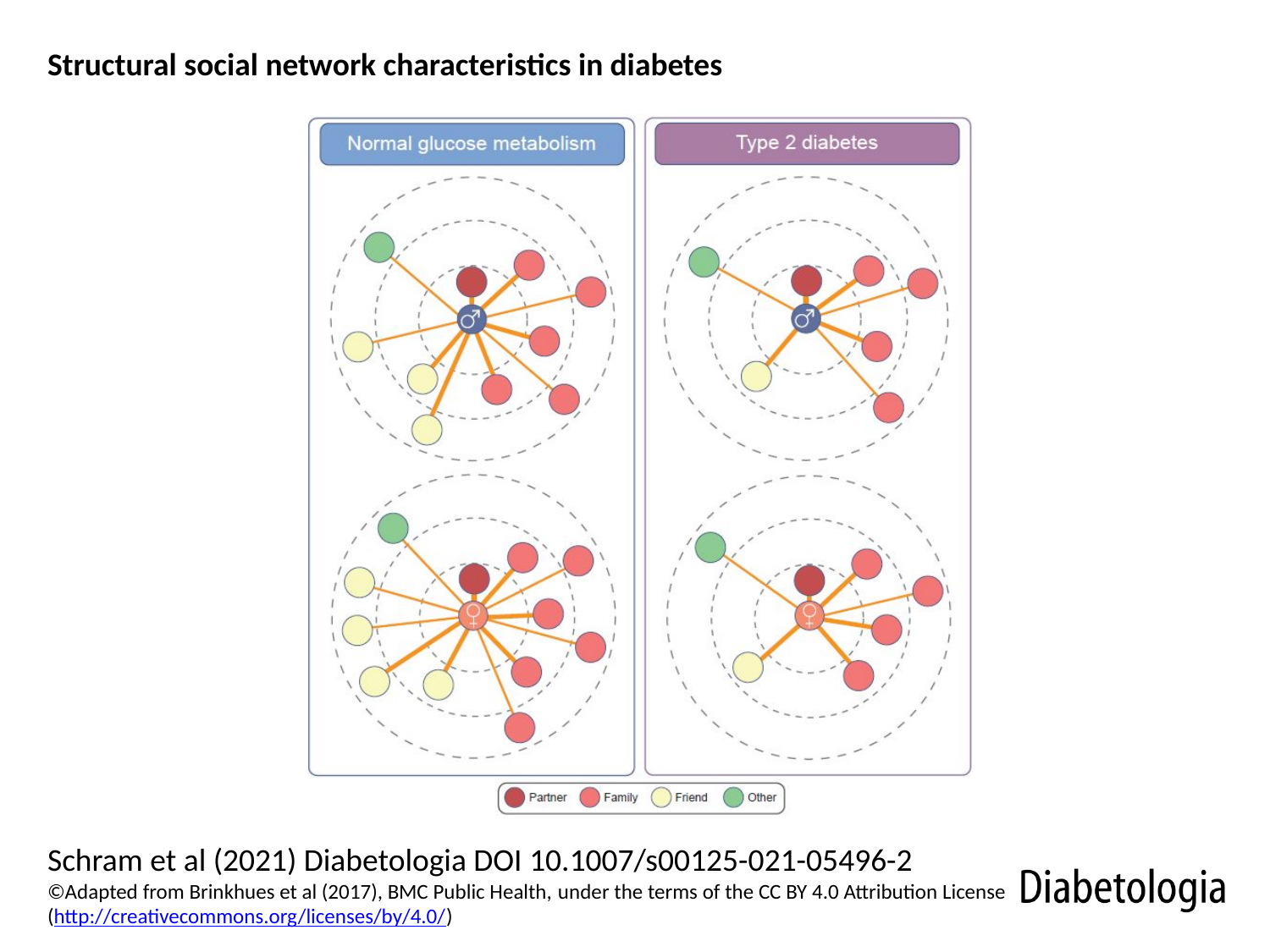

Structural social network characteristics in diabetes
Schram et al (2021) Diabetologia DOI 10.1007/s00125-021-05496-2
©Adapted from Brinkhues et al (2017), BMC Public Health, under the terms of the CC BY 4.0 Attribution License (http://creativecommons.org/licenses/by/4.0/)
